# Behavioral Activation and Depression Symptomatology: Longitudinal Assessment of Linguistic Indicators in Text-Based Therapy Sessions

**DOI:** 10.2196/28244

**Published:** 2021-07-14

**Authors:** Hannah A Burkhardt, George S Alexopoulos, Michael D Pullmann, Thomas D Hull, Patricia A Areán, Trevor Cohen

**Affiliations:** 1 Department of Biomedical Informatics and Medical Education University of Washington Seattle, WA United States; 2 Weill Cornell Institute of Geriatric Psychiatry Weill Cornell Medicine White Plains, NY United States; 3 ALACRITY Center Department of Psychiatry and Behavioral Sciences University of Washington Seattle, WA United States; 4 Talkspace New York, NY United States

**Keywords:** natural language processing, text analysis, behavioral activation, depression, digital interventions, mental health

## Abstract

**Background:**

Behavioral activation (BA) is rooted in the behavioral theory of depression, which states that increased exposure to meaningful, rewarding activities is a critical factor in the treatment of depression. Assessing constructs relevant to BA currently requires the administration of standardized instruments, such as the Behavioral Activation for Depression Scale (BADS), which places a burden on patients and providers, among other potential limitations. Previous work has shown that depressed and nondepressed individuals may use language differently and that automated tools can detect these differences. The increasing use of online, chat-based mental health counseling presents an unparalleled resource for automated longitudinal linguistic analysis of patients with depression, with the potential to illuminate the role of reward exposure in recovery.

**Objective:**

This work investigated how linguistic indicators of planning and participation in enjoyable activities identified in online, text-based counseling sessions relate to depression symptomatology over time.

**Methods:**

Using distributional semantics methods applied to a large corpus of text-based online therapy sessions, we devised a set of novel BA-related categories for the Linguistic Inquiry and Word Count (LIWC) software package. We then analyzed the language used by 10,000 patients in online therapy chat logs for indicators of activation and other depression-related markers using LIWC.

**Results:**

Despite their conceptual and operational differences, both previously established LIWC markers of depression and our novel linguistic indicators of activation were strongly associated with depression scores (Patient Health Questionnaire [PHQ]-9) and longitudinal patient trajectories. Emotional tone; pronoun rates; words related to sadness, health, and biology; and BA-related LIWC categories appear to be complementary, explaining more of the variance in the PHQ score together than they do independently.

**Conclusions:**

This study enables further work in automated diagnosis and assessment of depression, the refinement of BA psychotherapeutic strategies, and the development of predictive models for decision support.

## Introduction

Over 20% of adults in the United States have a mental illness [1]. Depression is among the most common mental health disorders: Over 19 million adults suffered major depressive episodes in 2019. Effective delivery of mental health services is a challenge for many reasons, including that individuals respond differently to therapy [2,3]. To maximize treatment benefits, mental health care providers must continually assess progress and adjust treatment plans [2]. From a research perspective, longitudinal information about known and hypothesized mechanisms of recovery is a prerequisite to the refinement of current interventions and can inform the development of new ones. Validated survey instruments exist to assess symptoms and other constructs relevant to therapy delivery and progress [4]; however, repeatedly filling out questionnaires places a burden on patients and providers, limiting the frequency with which these data can be collected. In contrast, using already available data created as part of routine care obviates the need for additional data collection. Additionally, it has been argued that subjective self-reports present potential limitations, for example due to cognitive and memory bias [5]; while careful scale design can alleviate these problems, objective, naturalistic measurements may be preferable.

### Behavioral Activation and Engagement

The behavioral theory of depression states that depressed individuals participate in fewer pleasant activities and derive less pleasure and feelings of accomplishment from such activities [6]; in other words, they exhibit reduced behavioral activation (BA). This phenomenon is self-exacerbating: Reduced activation represents a loss of positive feelings that further reduces activation. Neurobiological findings suggest that dysfunction of reward networks (especially in reward valuation, effort valuation, action selection, preference-based decision making, and reward learning) is a central process perpetuating depression [7,8]. For this reason, “reward exposure” aiming to induce BA has been thought to reactivate and retrain reward networks and improve depression [9]. BA therapies are therapeutic approaches based on the relationship between depressive symptomatology and engagement with pleasant activities. They aim to reduce depression symptoms by activating the reward system and have been shown to be as effective as learning-based therapies while being easier to understand for patients and easier to deliver for therapists [6]. An example is the streamlined, evidence-based psychotherapeutic strategy called Engage [7], which aims to systematically address disengagement from participation in pleasurable activities in a structured approach by incorporating reward exposure and addressing barriers in 3 behavioral domains: negativity bias, apathy, and emotional dysregulation. A recent randomized controlled trial showed that Engage is as effective as problem-solving therapy in treating late-life major depression, while having the advantage of being less complex; Engage required 30% less training time compared to problem-solving therapy [10].

To better understand the relationship between BA-based therapies and therapeutic response in depression, robust metrics of the underlying theoretical constructs that minimize reliance on patients’ and providers’ subjective reports are needed. Text-based therapy sessions provide a unique opportunity to develop such metrics because all language exchanged in these encounters is archived.

### Language as an Indicator of Mental State

Language reflects both conscious and subconscious thoughts and feelings [11-13]. Previous work has shown that depressed individuals use language differently than nondepressed individuals in a manner anticipated by cognitive theories of depression. For example, depressed individuals use more first-person singular pronouns (eg, “I,” “me,” “my”) than nondepressed individuals [14,15], indicating increased self-focused attention, a language use consistent with Pyszcynski and Greenberg’s [16] integrative model of depression. Depression has also been shown to be associated with a lack of social integration or social disengagement [17-19]. For this reason, Rude et al [14] anticipated a reduction in use of first-person plural pronouns (eg, “we,” “us”) in depressed college students but had too low a base rate to assess its impact in the sample available for analysis. Stirman and Pennebaker [19] found that suicidal poets used fewer first-person plural pronouns than nonsuicidal poets. Linguistic indicators of positive and negative affect differ in depression and have shown utility in social media–based predictive models of depression [14,20]. These findings are consistent with the emphasis on negative valence in Beck’s [21] influential depression theory. Finally, prior work has investigated content word usage by depressed individuals compared to control groups without depression. These include words related to sadness, as well as words related to somatic health concerns (health and biology words, with the biology category combining body, health, sexual, and ingestion words) [22,23]. Given these findings, the question arises whether variations in language use related to the behavioral theory of depression can be detected through natural language processing.

One approach to capturing emotional affect, linguistic style, and topics in written text is to calculate the percentage of words belonging to defined categories, such as positive affect words, pronouns, or words related to certain topics (eg, health or leisure). The Linguistic Inquiry and Word Count (LIWC) software package, a tool developed to study linguistic indicators of mental states, embodies this technique and was used to quantify relevant pronouns and affect words in the aforementioned work. As reviewed by Tausczik and Pennebaker [24], numerous experiments have validated the LIWC categories. However, while LIWC constructs such as “leisure” are related to the notion of activation, they do not provide a comprehensive account of how engagement might manifest in language. For example, categories of relevance to BA, such as the breadth of activities one engages in or the extent to which one derives a sense of accomplishment from setting and achieving activity goals, are not represented in the LIWC standard dictionaries.

Distributional representations of words learned from large amounts of electronic text can help construct comprehensive sets of terms similar to the curated sets used by LIWC to represent categories. Also known as semantic vectors or word embeddings, these representations are learned from text, with a typical approach involving first initializing random vectors of user-defined dimensionality and then iteratively updating them to make vectors for words appearing in similar contexts similar to one another. With neural embeddings, this is achieved by training a neural network model to predict the words surrounding an observed word and retaining some of the neural network weights after training to serve as word embeddings. Empath [25] is a tool designed to support rapid computer-assisted construction of user-defined term sets using such embeddings to find terms that are similar to an initial set of seed terms. Term sets constructed in this way have a strong correlation with the corresponding LIWC categories, which were constructed in a completely manual process. In essence, Empath’s approach uses distributional representations of words to identify similar terms to a set of seed terms based on their distributional statistics across a large text corpus. In this way, a small seed set of terms can be rapidly expanded to provide adequate coverage, with the expanded list provided to manual reviewers for pruning of those terms considered to be inconsistent with the category of interest. Empath’s vector representations are derived from a corpus of fiction. Though generally harder to come by, customized in-domain training corpora are known to produce better word representations in clinical domains [26].

For the current work, we developed a metric of BA, using distributional representations derived from a large corpus of naturally occurring language from online therapy chat messages (n=2,527,783) and characterized its relationship to indicators of depression severity. We hypothesized that linguistic markers of activation would be more frequent in milder depression than in severe depression and that longitudinal changes in these markers would reflect the trajectories of patients’ depression; patients who improve over time should also show an increase in BA. We further hypothesized that linguistic markers of BA would capture a separate, clinically meaningful dimension of depression symptomatology — namely, engagement in meaningful, rewarding activities — compared to the established linguistic indicators, which capture psychological manifestations of depression (self-focused attention or social integration [function word usage] and emotional tone) and content topics (sadness, health, biology words). Therefore, the BA metric should capture information beyond that reflected by established markers. We tested these hypotheses in the subset of messages from the time period where evaluations of the severity of depression were available for participants at regular intervals (n=1,051,025).

## Methods

### BA Lexicon

We developed a lexicon of related words collectively representing the construct of activation as used in BA. We constructed a set of 66 unique representative seed terms, informed by the Activation subscale of the Behavioral Activation for Depression Scale (BADS) [27], a validated instrument used to identify subjective engagement levels. The subscale consists of 7 questions, each aiming to capture a unique component of the construct. Seed terms were selected manually for each question in collaboration with GA, a clinician investigator with extensive experience in BA approaches (Table 1).

We expanded the sets of terms for the novel LIWC construct by using methods of distributional semantics, which generate vector representations of words from their distributional statistics in text, such that words occurring in similar contexts will have similar vector representations [28,29]. Specifically, we used the open-source Semantic Vectors software package [30-32] to train 100 dimensional word embeddings using the skipgram-with-negative-sampling algorithm [33] on a set of 2.5 million de-identified messages sent by Talkspace clients (>165 million total words). Embeddings were trained over 10 epochs, using a sliding window radius of 2 and a subsampling frequency threshold of 10^-5^. Words occurring fewer than 5 times in the corpus were excluded from training. This minimum frequency threshold is employed to restrict model consideration to those terms that occur in a sufficient number of contexts to inform a distributional representation and to constrain the number of vectors to save time (during nearest neighbor search) and disk space. We did not attempt to optimize this parameter but note that it is the default in the canonical implementation of the skipgram-with-negative-sampling algorithm [34]. For each seed term, we then added the 30 most related terms as determined by the cosine similarity between the seed term’s vector representation and the vectors for all other terms. We chose to add 30 because this number appeared to achieve high coverage while imposing a manageable workload for manual pruning. Note that stemming is not necessary, as this process will capture all forms of a word appearing in the training text, while preserving their semantic nuances; further, keeping all words appearing in the raw text ensures consistency between our dictionary and the texts to be assessed. For illustrative examples of similar words, see Table 2.

These lists of terms were manually filtered to remove irrelevant or inaccurate terms. Then, we solicited feedback and suggestions from GA. Feedback was incorporated at the seed term and manual filtering steps. The process was iteratively repeated until the lexicon was found to be to be satisfactorily inclusive and specific. Finally, the expanded lists of words, one per seed term, were combined to form 7 partially overlapping subconcepts according to Table 1, as well as one overarching “activation” category. In the overarching activation category, duplicate terms were removed to prevent double counting. We obtained a set of 1059 unique words, which represent the overarching idea of BA. The words originating from each of the 7 items in the activation subscale of the BADS yielded the subconcepts: *satisfaction* (227 words), *breadth* (341 words), *decisions* (205 words), *accomplishment* (154 words), *long-term* planning (240 words), enjoyment of *effort* (342 words), and *structure* (216 words). The complete term sets are available in the accompanying online repository [35]. LIWC was then used to measure the frequency of words belonging to each construct as a metric of patient engagement in BA.

**Table 1:** Seed terms derived by the authors from the individual questions in the “Activation” subscale of the Behavioral Activation for Depression Scale (BADS).

Item	Brief name assigned by the authors	Derived seed terms^a^
I am content with the amount and types of things I did.	Satisfaction	Accomplish, achieve, satisfaction, satisfied, enjoy, content, contentment, accomplishment, love, proud, inspired, inspiring, enthuse, affirm
I engaged in a wide and diverse array of activities.	Breadth	Activity, active, participate, involved, event, powerlifting, water coloring, exercise, sport, basketball, restaurant, hobby, craft, art, music, instrument, piano
I made good decisions about what type of activities and/or situations I put myself in.	Decisions	Decision, planning, plan, contest, competition, opportunity, chance, spontaneous, whim, spur, attentive, affirm, commit, focus
I was an active person and accomplished the goals I set out to do.	Accomplishment	Goals, accomplish, progress, goal, achieve, effort, content, contentment, accomplishment, proud
I did things even though they were hard because they fit in with my long-term goals for myself.	Long-term	Goals, progress, goal, effort, planning, plan, challenge, attentive, birth, commit, change, invest, life, payoff, benefit
I did something that was hard to do but it was worth it.	Effort	Effort, enjoy, excited, energized, energizing, love, contest, competition, challenge, chance, fun, enthusiastic, inspired, inspiring, enthuse, event, affirm, commit, change, focus, fuel, invest, invigorate
I structured my day’s activities.	Structure	Goals, progress, goal, planning, plan, structure, attentive, event, routine, schedule, regular

^a^There are 104 total words in the right column, including duplicates (eg, “goals” appears in accomplishment, long-term, and structure) for a total of 66 unique terms.

**Table 2:** Examples of seed terms and similar terms with corresponding similarity score, calculated by computing the similarity between word vectors.

Seed terms and similar terms^a^		Similarity score
**Proud**		
	Accomplished	0.729
	Accomplishment	0.679
	Accomplishments	0.673
	Impressed	0.667
	Prouder	0.663
	Gussied	0.646
**Active**		
	Inactive^b^	0.659
	Activity	0.633
	Powerlifter	0.607
	Motivated	0.605
	Mighy	0.600
	Intramural	0.592
**Decision**		
	Decisions	0.863
	Choice	0.841
	Deciding	0.723
	Hyphenating	0.697
	Choices	0.693
	Decide	0.671
**Goal**		
	Goals	0.865
	Attainable	0.761
	Achievable	0.740
	Acheive	0.725
	Aim	0.722
	Accomplish	0.717
**Commit**		
	Committing	0.828
	Committed	0.788
	Babydaddy^b^	0.708
	Committ	0.708
	Commitment	0.706
	Sucide^b^	0.682
**Effort**		
	Efforts	0.729
	Concerted^b^	0.718
	Valiant	0.690
	Handsomeness^b^	0.687
	Timeand^b^	0.662
	Independents^b^	0.648
**Routine**		
	Routines	0.874
	Schedule	0.708
	Nighttime	0.698
	Regimen	0.691
	Rhythm	0.682
	Schefule	0.682

^a^Terms were extracted from our chat message corpus and thus include common typographical errors.

^b^Words that were removed in the filtering process.

### LIWC

The LIWC [24,36] software package, developed by Pennebaker and his colleagues over the past 2 decades, was used for linguistic analysis. LIWC derives features from narrative text by counting the number of words in a text that correspond to categories in the LIWC lexicon (or dictionary), with categories defined by lists of words that fall into them. LIWC returns the percentage (or proportion) of words in a text that correspond to each category. For example, consider the following excerpt from an interview with singer, songwriter, and poet Leonard Cohen:

When I speak of depression, I speak of a clinical depression that is the background of your entire life, a background of anguish and anxiety, a sense that nothing goes well, that pleasure is unavailable and all your strategies collapse.

37

This excerpt is 40 words long, and the words “depression” (n=2), “anguish” (n=1), and “anxiety” (n=1) fall into the LIWC negative emotion category. Therefore, LIWC returns a percentage score of 10% (100*4/40) for this category. Other categories are measured similarly by estimating the frequency with which words they include occur in a unit of text. However, LIWC also includes a set of composite categories that are derived by combining individual categories. As negative and positive affect are both potentially informative, for parsimony, we considered the composite emotional tone variable, which combines the positive and negative emotion categories. A high tone score indicates a predominance of positive over negative emotion words, and a low score indicates the opposite. A score of 50 indicates a balance between positive and negative affect [36].

Additionally, we measured the usage rates of first-person singular pronouns, first-person plural pronouns, and words belonging to the content categories health, biology, and sadness. Finally, we measured linguistic indicators of BA by counting the number of BA lexicon words overall and in each subcategory in every patient’s messages.

### Data

This work utilized de-identified chat messages sent during routine online therapy, collected for a previously reported study by Hull et al [38]. Clients took part in messaging therapy conducted by a licensed, certified clinical professional via the Talkspace online platform over 12 weeks. The platform provides a paid service open to all, and the service may be covered by some insurers. Therapists and clients converse via written, asynchronous messaging on the platform, and therapists utilize a range of therapeutic strategies. The platform also allows users to send video and audio messages, though these were not used in the current work. Only client messages collected during the course of therapy were used in this study. Participants completed the Patient Health Questionnaire 9-item (PHQ-9) questionnaire at baseline as well as every 3 weeks during therapy. The PHQ-9 is a validated self-report questionnaire commonly used to assess depression severity, scored on a scale of 0-27 [4]. For further details on the platform, data collection process, and study population, see Hull et al [38].

The participants (N=10,718) were young (≤35 years old: 8014/10,142 [576 age values were missing], 79.02%; none younger than 18 years), educated (Bachelor’s degree or higher: 6871/9169 [1549 education values were missing], 74.94%), and mostly female (8340/10,571 [147 gender values were missing], 78.90%). Data on race and ethnicity are not systematically collected by the digital platform and are missing for most participants. There were a total of 24,387 PHQ-9 assessments with corresponding messages, with 37.65% of participants (4035/10,718) only completing the baseline assessment, 24.50% (2626/10,718) completing 2 assessments, 18.31% (1962/10,718) completing 3 assessments, 9.69% (1038/10,718) completing 4 assessments, and 9.86% (1057/10,718) completing 5 assessments. The mean baseline PHQ score was 13.36 (SD 4.96) and did not significantly vary with the total number of assessments completed. The mean end PHQ score was 10.80 (SD 5.83) and was significantly lower the more assessments were completed. Patients participated in chat conversations throughout the study period, as well as in the 3 weeks leading up to the baseline assessment in some cases, which were included when available (weeks –3 through –1). Messages were aggregated by concatenating them (ie, combining messages in sequence), creating a single “document” as the unit of analysis. For studies 1 and 2, each PHQ score was used to label the pooled messages from the period on which the questionnaire asks respondents to reflect (the 2 previous weeks). PHQ-9 questionnaires were filled out at the beginning of weeks 0, 3, 6, 9, and 12. For study 3, messages were pooled by week (starting with week –3), and each series of (up to) 15 datapoints has one trajectory label. On average, participants had 7.4 weeks of messages and wrote 770 words per week; patients completed 2.2 assessments on average and wrote 2133 words per completed assessment. At baseline, the number of words written did not vary significantly with depression severity (*P*=.33). There were 79,096 weeks of messages and 23,950 PHQ assessments with messages. For discussion of the relationship between demographic and engagement factors and treatment outcomes, please see Hull et al [38].

#### Trajectory Labels

Based on patients’ longitudinal PHQ-9 and General Anxiety Disorder-7 (GAD-7) scores, Hull et al [38] clustered patients using latent growth modeling and assigned the following labels to the 6 trajectory groups that emerged: Acute Recovery, Recovery, Depression Improvement, Anxiety Improvement, Chronic, and Elevated Chronic. The middle 2 categories appeared to capture patients who improved in some symptoms but not others. Additionally, improvements in PHQ (or lack thereof) were less clear than in the other groups. Because the individuals in these groups are thus outside the simple definitions of depression “improvement” and “nonimprovement,” they were not included in analyses of binary improvement status. A subset of 6760 patients was used for trajectory analysis. Of these patients, which Hull et al [38] identified as strictly “improving” or “nonimproving,” 47.2% (3189/6760) improved (classified as Recovery or Acute Recovery), and 52.8% (3571/6760) did not improve (classified as Chronic or Elevated Chronic).

This study focused on ascertaining the utility of linguistic markers to predict depression symptom improvement only; therefore, when using trajectories, we simplified trajectories into a binary “improvement” label, with the 2 recovery classes in the improvement group and the 2 chronic classes in the nonimprovement group.

### Statistical Analysis

#### Study 1: Association of Linguistic Markers With PHQ

To validate the basic premise of LIWC and the BA concept, we investigated the relationship between linguistic markers and PHQ-9 scores using the (up to) 5 measurements of linguistic indicators with corresponding PHQ-9 scores per patient. For this analysis, each pair of PHQ-9 assessment and corresponding message log was treated as a data point. We first determined whether the established LIWC metrics as well as our novel BA metric are statistically significantly different between patients with different depression symptom severity. Severity was defined by the clinical depression symptomatology groups used by the PHQ scoring system: minimal (PHQ ≤4), mild (PHQ=5-9), moderate (PHQ=10-14), moderately severe (PHQ=15-19), and severe (PHQ ≥20). Further, the average difference in each linguistic marker for each unit difference in PHQ score was determined using mixed effects linear regression, treating the patient identity as a random effect.

#### Study 2: Utility of BA Subconstructs

Each question of the BADS activation subscale aims to capture a distinct dimension of the theoretical construct. To determine the difference between the components and the potential clinical value of the subcomponents compared to pronoun usage, affect measures, and the overall BA concept, we conducted regression analyses on combinations of different variable subsets. For each analysis, we determined the variance explained by each subset of predictors in a mixed effects model with PHQ-9 score as the outcome, treating participant identity as a random effect. Predictors were combinations of (1) subsets of the established LIWC variables (first-person singular pronouns, first-person plural pronouns, emotional tone, or all 3; sadness, health, biology, or all 3) and (2) subsets of the BA variables (the overall construct, each of the 7 subconstructs, all 7 subconstructs, and all 7 subconstructs plus the overall construct). Comparing the amount of variance explained (R^2^) between baseline models and models that include additional variables yields insights into the extent to which the added variables provide further information. However, chance associations alone can increase R^2^ even if variables provide little usable additional information; therefore, we additionally determined the Akaike information criterion (AIC), which penalizes model fit in response to model complexity. A nonincreased AIC in conjunction with an increased R^2^ should therefore signal that added variables contained new information.

#### Study 3: Association of Linguistic Markers With Patient Trajectories

To determine the association between different linguistic indicators and outcome, mixed effects linear regression analysis was used to compare the rates of change of the variables over time between patient trajectories (whether patients were improving [ie, classified as Recovery or Acute Recovery] or nonimproving [ie, classified as Chronic or Elevated Chronic]).

We compared the average change in each variable per 1-week difference (regression slope). For this analysis, messages were aggregated by week, yielding a time series with up to 15 data points for each patient. Thus, we calculated how PHQ scores and linguistic indicators changed with time in the improving and nonimproving groups, controlling for the within-patient dependency of samples. Specifically, for each of the 2 groups, we fitted a mixed effects linear regression model of the following form:

Y_i j_ = β_0_ + β_1_X_i j_ + γ_0i_ + γ_1i_X_i j_ + ε_i j_

Where *Y_i j_* is the variable of interest measured for participant *i* in week *j* (eg PHQ, activation, or satisfaction), *X_i j_* is the week number, β_0_ and β_1_ are the fixed effect (time) parameters, and γ_0i_ and γ1i are the random effects (participant ID) parameters. Calculations were done using the statsmodels package in Python [39].

## Results

### Study 1: Relationship Between Linguistic Indicators and PHQ Scores

**Figure 1:**
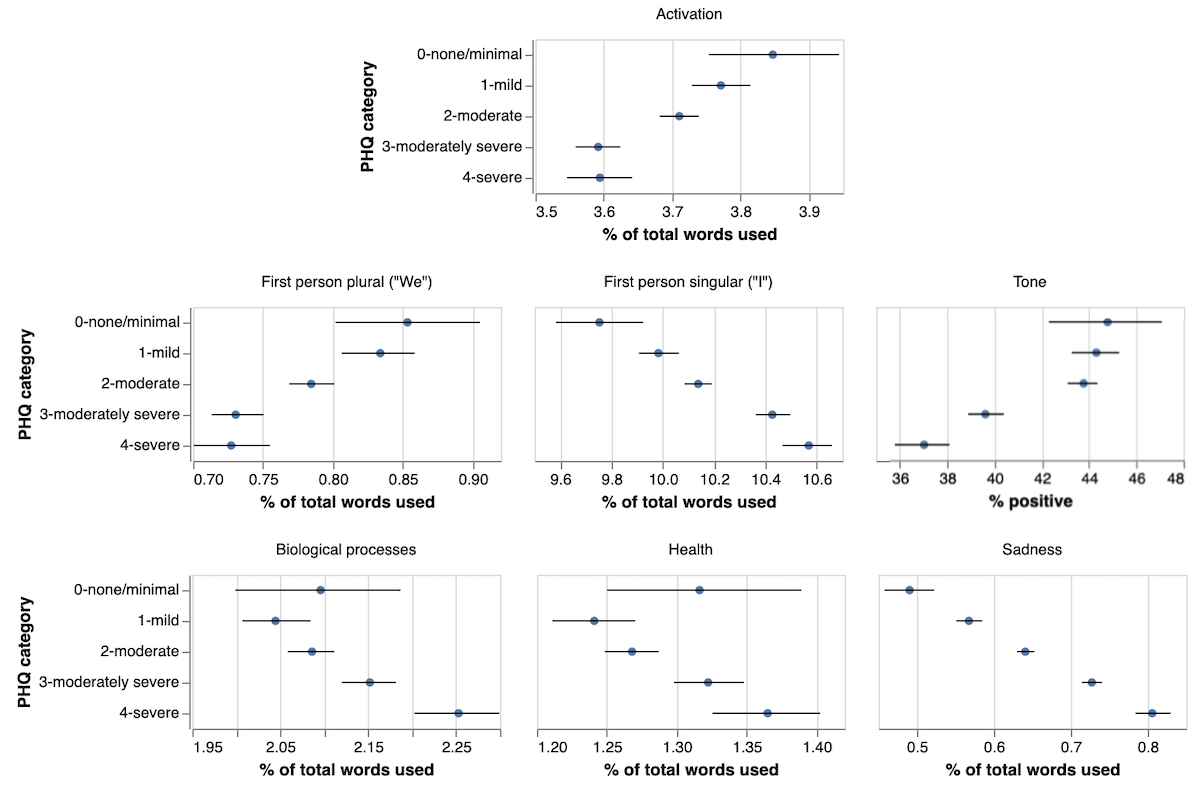
Bootstrapped 95% CIs of the mean of each Linguistic Inquiry and Word Count (LIWC) measure by depression symptom severity category at baseline: minimal (Patient Health Questionnaire [PHQ] ≤4, n=393), mild (PHQ=5-9, n=1865), moderate (PHQ=10-14, n=4109), moderately severe (PHQ=15-19, n=3002), severe (PHQ ≥20, n=1331).

Using LIWC to measure the percentage of words belonging to the overall activation construct (including all terms related to any of the subconstructs), the average level of activation across the baseline chat logs was 3.66 (SD 0.89) and varied significantly with the depression symptom severity category (Figure 1), as did the LIWC emotional tone measure and the LIWC pronoun measures (first-person singular and first-person plural). Less depressed individuals used more “we” pronouns and fewer “I” pronouns. All individuals expressed more negative affect than positive (tone <50), with the most depressed individuals exhibiting an emotional tone balance most extremely tipped towards negative affect (lowest scores). The topic-related word categories were also significantly different between severity groups, with sadness having the most pronounced differences between groups. The health and biology categories appear to show increased usage in more depressed individuals but have remarkably large confidence intervals for the least depressed group (none/minimal). Higher overall BA levels were detected for lower depression levels, indicating that patients with more severe depression symptoms discussed activities and associated feelings of enjoyment and reward less than their less-depressed counterparts.

### Study 2: Utility of Subconstructs

**Figure 2:**
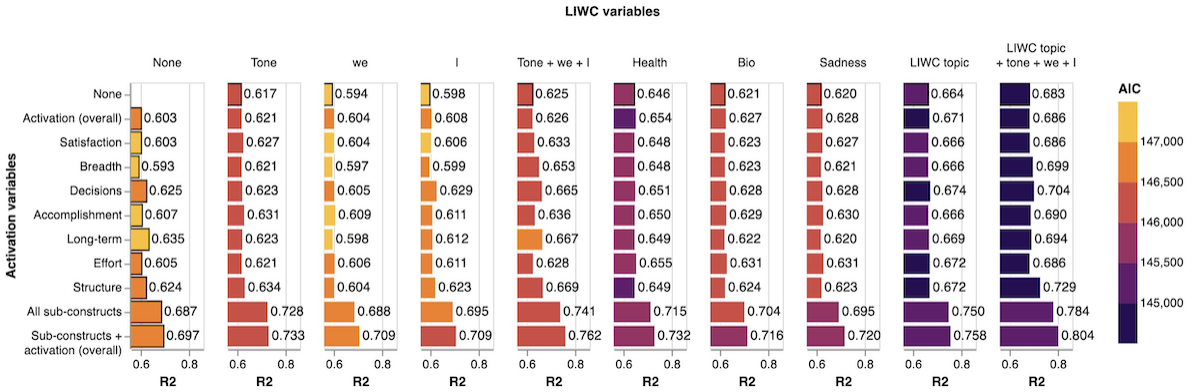
Variance explained (R2) by each subset of variables in a mixed effects model with Patient Health Questionnaire (PHQ) score as the outcome. Compare columns to the left column (baseline) for the increase in R2 due to standard Linguistic Inquiry and Word Count (LIWC) variables compared to activation variables alone; compare rows to the top row (baseline) for the increase in R2 due to activation variables compared to standard LIWC variables alone. Darker AIC colors indicate better fit.

The variance in the overall PHQ score explained (R^2^) by the 109 models fitted to all possible combinations of the LIWC and BA variable sets is shown in Figure 2. The amount of variance explained for each baseline model is shown as bars with strokes, and comparison values are shown without. Darker colors indicate better fit as measured by the AIC. At baseline, emotional tone is more informative than “I” or “we.” The health category is the most informative topic category; the 3 topics together explain more variance than the tone and pronoun variables together and also have better fit. All LIWC variables together explain the most variance without detracting from model fit. Of the activation subconstructs, the decision, long-term planning, and daily structure components are most informative; again, all variables together explain the most variance. Including tone added more to satisfaction, breadth, accomplishment, and effort than to decisions, long-term planning, and daily structure. LIWC package constructs alone (tone, I, we; content topics) accounted for 68.3% of the variance, while the combination of our newly created BA subconstructs plus total score alone accounted for 69.7% of the variance. The highest R^2^ of 80.4% was achieved by the model that included all variables: emotional tone, function words (I, we), all 3 topic categories, and all 7 subcomponents of activation, along with the overall activation level. Interestingly, including the overall BA concept along with the BA subconstructs appeared to improve both R^2^ and fit compared to the subconstructs alone.

### Study 3: Relationship With Patient Trajectories

**Figure 3:**
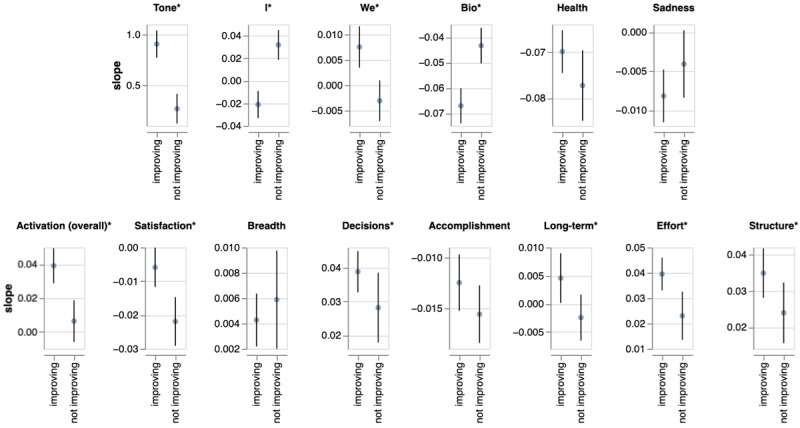
Regression coefficients and corresponding 95% CIs of the mixed effects models (ie, the average change in the given variable for each treatment week). *P<.05.

Figure 3 shows the average change in each linguistic marker per week in the improving and nonimproving groups. Several linguistic indicators showed average amounts of change over time that were significantly different between the 2 groups.

Of the established LIWC markers, emotional tone, first-person singular pronouns, first-person plural pronouns, and biology words were different between groups. Interestingly, biology word usage decreased less in the nonimproving group than in the improved group, while health word usage decreased more in the nonimproving group. Sadness was reduced in both groups over time, with a larger change in the improving group, though the difference between groups was not statistically significant.

**Figure 4:**
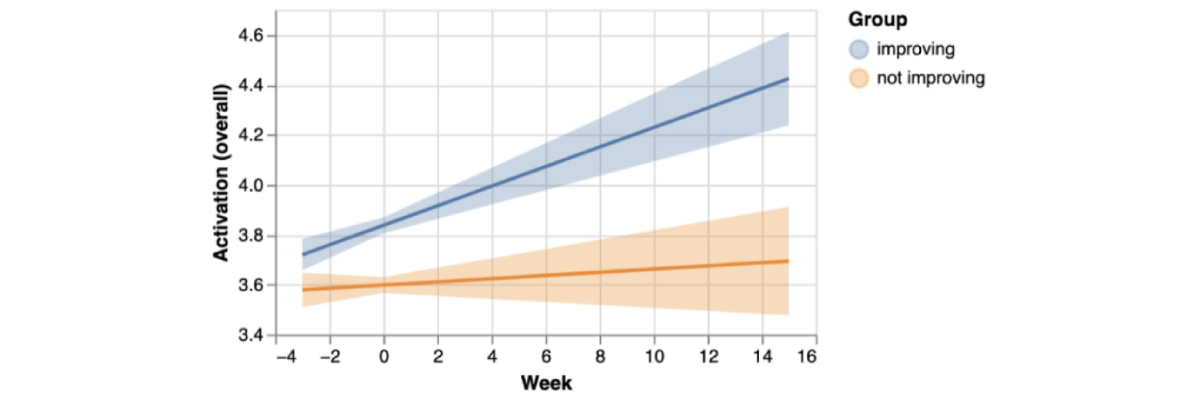
Fixed effects of the fitted linear mixed effects models for the improving and nonimproving groups for activation (overall). Improving: activation = 3.837 + week * 0.039; nonimproving: activation = 3.598 + week * 0.006.

Of the linguistic markers of BA, the markers for satisfaction with activities and rewarding effort had the most pronounced difference between groups, along with the overall activation marker. The fitted fixed effects models are shown in Figure 4. Neither the breadth of activities discussed nor mentions of feelings of accomplishment were different between groups.

## Discussion

In this work, we developed an approach to measuring BA from patients’ natural language. We demonstrated that the resulting metric, like scores collected with the validated PHQ-9 depression symptom questionnaire, can discriminate between patients on positive and adverse trajectories. Furthermore, we demonstrated that activation can be measured in terms of its distinct, clinically meaningful subconstructs, each complementary to established linguistic indicators (affect, pronouns, content categories), and has potentially different uses in future research and applications. Additionally, we demonstrated that established linguistic indicators obtained using LIWC are associated with changes in depression symptomatology as measured with the PHQ in a large sample of online therapy clients.

At baseline, linguistic markers captured the depression severity experienced by patients in this study. The established LIWC markers of affect, pronouns, and topic categories were significantly different between PHQ-9 severity groups. Remarkably, sadness and the indicator of self-focus showed nearly identical patterns across groups. Though depression without sadness does occur, sadness is perhaps the most famous marker of depression. The agreement between first-person singular pronouns and sadness confirms the theory of self-focused attention and provides further support for the measurement of self-focus using LIWC. Further, even at baseline, BA was markedly different between depression severity groups. The presence of this association at baseline provides support for the connection between activation and depression symptoms that is the basis for BA therapies.

Notably, not all variables can differentiate all depression severity groups. For example, tone appears to be clearly different between the moderately severe and severe groups at baseline, while activation is not. Conversely, mean activation is different between moderately depressed individuals and those with only minimal symptoms, while tone was not. That not all variables were different between all groups may be related to the polymorphous clinical presentation of depression; patients experience different combinations of symptoms to varying degrees [40]. The heterogeneous presentation of depression suggests that any single metric can only partially capture the clinical severity of depression and underscores the importance of measuring multiple aspects of depressive symptomatology. Combining several complementary markers, each carrying some unique information, can maximize predictive power by capturing different dimensions of depressive psychopathology.

The mechanisms underlying improvement due to BA therapies are not sufficiently well understood. A comparison of different BA approaches found that most result in similar benefits, even though they include slightly different protocols, suggesting that some elements of the various activation approaches may be unnecessary [6]. By investigating the individual components of the activation subscale of the BADS in isolation, we showed that each contributes further information: Considering the individual parts, rather than just the overall idea of activation, explains more variance in regression models without deteriorating model fit (worse model fit might be expected if more variance was explained merely because more variables were included). This finding suggests that the features capture distinct dimensions of activation and that some dimensions may be more important than others. For example, the idea of breadth (corresponding to the BADS item “I engaged in a wide and diverse array of activities.“) was least predictive of patient improvement over time.

Manos et al [41] previously showed via factor analysis that a modified version of the breadth item (“I engaged in many different activities”) should be retained in a short version of the BADS. However, the current work interpreted the item as capturing activity diversity specifically, counting ideas such as “exercise,” “restaurant,” and “instrument,” similar to the Pleasant Events Schedule (PES) questionnaire. The PES asks respondents for the number of times they participated in activities on a list of 320 options in the past 30 days, including “Playing baseball or softball,” “Going to a restaurant,” and “Playing a musical instrument” [42]. Manos et al [41] pointed out that PES may not represent the key functional activities of every respondent and therefore may not accurately capture activation. Their rewording of the item removes some of the focus on activity diversity. Our findings support the idea that focusing on activity diversity may not be beneficial for the identification of linguistic markers of BA. Future work might modify the dictionary created here to remove the specific named activities. Further research may also grant insights into opportunities to refine treatment interventions and protocols. For example, it may be prudent to explore modifying BA therapies to remove any emphasis on activity diversity and focus on the frequency of patients’ favorite activities.

Our analysis shows that emotional tone and first-person pronouns (singular and plural) are strongly associated with patient trajectory, consistent with prior work. Both the improving and nonimproving groups shifted to a more positive tone over the treatment period (positive, nonzero slope), which may be an effect of participating in therapy — participants may be focusing on solutions rather than problems in conversations with their therapists. While the sadness category was also strongly associated with patient trajectories, the effect was less pronounced than may have been expected, considering that feelings of sadness are common in depression. Surprisingly, the health topic decreased in usage more in those not on a positive trajectory than those who were, while the “biological processes” topic group decreased more in the improving group. The biological processes topic group contains health, ingestion, sexual, and body words. That patients on the path to recovery discuss these topics less as time passes may indicate that they experience fewer somatic symptoms.

In addition to the overall BA construct, we found several subconstructs strongly associated with patient trajectory. Satisfaction, decisions, long-term planning, effort, and structure all showed significant differences between the improving and nonimproving groups (*P*<.05). Of all activation subconstructs, breadth and accomplishment were most similar between these groups. However, the addition of breadth to the established LIWC markers resulted in more variance explained without reducing goodness of fit. In fact, breadth was the third most informative single activation construct when included alongside all topic and pronoun variables and tone. In other words, while breadth alone is not explanatory, it is informative in the context of other predictors such as emotional affect. This observation may indicate that discussing activities without positive feelings is not indicative of improvement; activities must also be perceived as rewarding.

Tausczik and Pennebaker [24] first began developing and using LIWC in the 1990s. Counting words belonging to semantic and syntactic categories is simple yet effective, as demonstrated in countless experiments across several fields [14,20,22,24,43,44]. A detailed review of LIWC applications is available elsewhere [24]. Despite this extensive existing work utilizing LIWC, sample sizes have historically been small: The largest sample size in the meta-analysis by Edwards et al [15] of first-person singular pronoun use in depression was 966. Containing chat conversations from over 10,000 individuals over almost 3 years, with over 74 million words, our dataset of naturally occurring language is considerably larger than those used in previous experiments validating the relationship between LIWC variables and depression. Our results provide further validation of the LIWC tone- and pronoun-related variables in the context of scores from a validated, standardized instrument for measurement of symptomatology. Changes in established LIWC variables are consistent with case-control differences demonstrated in prior research [14,19,20], with tone and first-person plural pronouns increasing as symptoms decrease and first-person singular pronoun usage decreasing with improvement in symptoms. Therefore, we showed that these metrics indeed reflect depression symptom status in this dataset, providing further strong support for their relationship with depression. However, in the context of the current data, BA variables explained more of the variance in the PHQ data than pre-existing LIWC constructs.

### Limitations

Reliance on the PHQ-9 is a limitation because this instrument focuses exclusively on symptoms of depression. Future studies may use instruments assessing well-being and social adjustment.

The trajectories used to categorize patients as improving or otherwise were assigned via unsupervised learning and do not directly correspond to the total change in PHQ over the course of treatment. Rather, they account for the entire series of depression and anxiety scores over the treatment period. A participant may have an absolute decrease in PHQ score (eg, 16, 18, 15, corresponding to a total change in PHQ of 1 point between the beginning and end of treatment); however, the patient may not be experiencing clinically meaningful progress. Thus, depending on the entirety of the PHQ-9 as well as GAD-7 scores over the treatment period, the model may not assign an “improvement” category for such a patient. Many patients only had 1 or 2 scores, and some participants with identical sets of scores were assigned to different groups. However, mixed effects linear regression confirmed that depression symptoms as measured by PHQ-9 scores improve significantly more for patients in the improving group than for patients in the nonimproving group. Thus, we believe that the categories are sufficiently accurate for our purposes. While we excluded the “gray area” trajectories of Depression Improvement and Anxiety Improvement, it is worth noting that analyses were repeated with these included, and results, though less clear, were not different and our conclusions held; this is the expected result of introducing additional noise. Another potential limitation of the trajectories is that even the “nonimproving” group showed a slope in PHQ that was significantly different from 0; we believe that this is an effect of unsupervised learning (ie, the trajectories clustered into groups labeled as “chronic” are distinct from the other clusters, but not necessarily entirely without improvement) coupled with the fact that therapy and just the simple passage of time (regression to the mean) is at least somewhat effective for most people. A truly “nonimproving” control group is therefore difficult to carve out in this dataset, possibly because such people represent a minority of participants and were thus not assigned a separate cluster by the unsupervised approach. Future work on this dataset may consider using the more granular patient trajectories if the degree of improvement or exacerbation is of interest. For example, a patient may be responding well (“remission”), but there may be room for improvement (“acute remission”); alerting providers to this situation could result in additional efficiency of care.

The lexicon of words collectively representing activation developed in this work was based solely on the messages in this dataset. This approach ensured that the concept represents language usage in our specific study population. While domain-specific texts generally work better for distributional semantics than more general corpora, they may also result in artifacts with limited generalizability. Our study population was predominantly young (8014/10,718, or 74.77%, were 35 years old or younger) and, considering that they used a paid online therapy service, presumably financially stable; in addition, 2679 (2679/10,718, 25.00%) were residents of California or New York, and 8340 (8340/10,718, 77.81%) were female. This striking gender imbalance is consistent with previously reported gender imbalances in both online and face-to-face therapy. For example, Chester and Glass [45] reported that 70% of online therapy clients are female, and a recent comparable online mental health service in Australia [46] reportedly had 72% female clients. Sagar-Ouriaghli et al [47] reported that women are 1.6 times more likely to receive any form of mental health treatment than men. Nevertheless, the makeup of our study population must be considered in future applications of our results. Geographically and demographically diverse groups may significantly differ in their word choice and usage; as a result, the lexicon may not be generalizable to other groups.

Word count approaches such as the one employed by LIWC have limitations. While most modern natural language processing methods account for negations, counting words does not. LIWC has a separate “negation” category, but it only considers single words and thus does not assign negation statuses to individual concepts. For example, describing having planned the day’s activities and *not* having planned the day’s activities would both count towards our *structure* concept in the same way. Because both statements reflect that the patient engaged with the idea of planning their day’s activities, this is arguably a minor limitation. Still, our results indicate that context is more important for some concepts than others; for example, breadth is only informative when considered alongside other markers such as emotional affect to contextualize it. In our future work, we plan on utilizing approaches that can account for negations.

A potential limitation is that the therapy sessions in this work did not specifically use BA therapy. However, BA is a common pathway of many therapies, which through a variety of interventions, increase exposure of depressed patients to meaningful, rewarding experiences.

An important factor to consider when proposing such automated analyses is the degree to which passive monitoring of therapist-client communication for the purpose of measuring BA may be construed as invasive or intrusive. While we did not directly engage with patients in the current work, we note that our recent work in the suicide prevention domain provides some indication that passive monitoring of this sort may be acceptable to patients when conducted by a trusted party, with 68% of survey participants indicating that the automated analysis of personalized web search data for suicide prevention would be acceptable provided this triggered minimally invasive interventions (such as connection to a support network or therapist) only [48].

Another limitation of the study is the absence of qualitative review that could examine the accuracy of our ratings.

### Future Work

An additional implication of the potentially causative mechanism of activation is that it should occur before symptom improvement. While more direct measures of sentiment and mindset reflect an individual’s current thoughts and feelings, “activity” topic analysis may reveal long-term dimensions of patient trajectory. Discussing activities, plans for activities, or even avoidance of activities shows that a patient engages with the ideas surrounding BA and may indicate that a patient is moving towards or already part of a positive feedback loop of self-perpetuating improvements. Consequently, one may expect metrics of activation to predict longer-term changes more accurately than word use analysis, which may be confounded by the mood of the moment, and thus capture a separate and clinically meaningful dimension of symptomatology and treatment success.

Our results show that the BA subconstructs of *decisions* (independent decision making), *long-term* (planning for the long term and acting accordingly), and *structure* (structuring daily activities) are parallel in terms of the amount of variance they explain. Let us call these *activities*. Similarly, *satisfaction*, *accomplishment* (a sense of having accomplished something), and *effort* (enjoyment due to effort exerted) are similar to each other. Let us call these *reactions*. Activities alone were more explanatory of improvement than reactions alone; in other words, activities are explanatory even without considering emotional tone. Adding tone increased the amount of variance explained for by reactions to the levels for activities alone. In other words, reactions are more informative when considered alongside tone than without, whereas adding tone makes comparatively little difference for activities. A possible explanation is that merely discussing feelings of satisfaction, or lack thereof, may not correspond to improvement. However, engaging in long-term planning, scheduling activities, and structuring routines does correspond to symptom improvement. Mentions of being satisfied may only be triggered positively once the positive feedback loop is set in motion. Conversely, this pattern is in agreement with the helplessness theory [16,49], which states that experiencing negative consequences to reward-seeking behavior perpetuates the avoidance phenomenon and therefore, continuing depression symptoms, by supporting a negative outlook on the future. Therefore, it requires additional information about the patient’s tone to be informative of current symptom severity. Therefore, an opportunity for future work is to investigate the temporal relationship between symptom improvement and the constructs in the activities group compared to those in the reactions group. One may be more useful than the other to predict long-term changes. Ascertaining the timeline of changes in these different activation metrics relative to patient trajectories is a prerequisite to the translation of linguistic indicators into clinical insights.

Recently, a broad range of sophisticated natural language processing techniques has gained traction. Our work here demonstrated that theme and affect analysis conducted via established, straightforward methods such as word counting both reflect and potentially predict depression status. It is plausible that more advanced methods may surpass those used here. Consequently, future work is set to include a deep neural network model trained on GoEmotions [50], a large corpus of social media posts annotated for the extent to which they express a set of emotion categories [51].

Having validated the linguistic indicators of depression and activation presented here, the question of how to incorporate them effectively into care processes remains. Predictive analytics solutions must be operationalized effectively to improve health outcomes. Accordingly, future work should focus on testing and iteratively refining these measures as part of care delivery. For example, metrics of depression symptom severity and BA may be automatically extracted from patient messages on virtual chat therapy platforms and used to guide the therapist in recognizing whether a patient responds well to therapy or if a change in direction is appropriate.

### Conclusion

This work makes several key contributions. First, we devised a computational method to automatically assess theoretical constructs of BA from patient language. Second, building on prior work demonstrating that activation has a close relationship with depression scores, we demonstrated this new metric reflects depression symptom severity. Third, we validated established linguistic markers of depression in a large corpus of naturally occurring language collected as part of psychotherapy sessions, presenting differences between participants with low and high PHQ-9 scores. Fourth, we showed that both the well-established LIWC measures as well as the novel BA measures have utility in predicting longitudinal patient trajectories. Finally, we demonstrated that our metrics of the individual subconstructs of BA capture distinct dimensions of the underlying mechanisms and may lend themselves to unique clinical insights. This work therefore enables further work in automated diagnosis and assessment of depression, as well as refinement of BA psychotherapeutic strategies.

## Abbreviations

AIC: Akaike information criterion
BA: behavioral activation
BADS: Behavioral Activation for Depression Scale
GAD-7: General Anxiety Disorder-7
LIWC: Linguistic Inquiry and Word Count
PES: Pleasant Events Schedule
PHQ-9: Patient Health Questionnaire 9-item
